# Rabbit Hepatitis E Virus Infections in Humans, France

**DOI:** 10.3201/eid2307.170318

**Published:** 2017-07

**Authors:** Florence Abravanel, Sébastien Lhomme, Hicham El Costa, Betoul Schvartz, Jean-Marie Peron, Nassim Kamar, Jacques Izopet

**Affiliations:** Centre de Physiopathologie de Toulouse Purpan, Toulouse, France (F. Abravanel, S. Lhomme, H. El Costa, N. Kamar, J. Izopet);; Centre Hospitalier Universitaire de Toulouse, Toulouse (F. Abravanel, S. Lhomme, J.-M. Peron, N. Kamar, J. Izopet);; Centre Hospitalier Universitaire de Reims, Reims, France (B. Schvartz)

**Keywords:** hepatitis E virus, HEV, rabbit hepatitis E virus, viruses, hepatitis, hepatitis E, infections, chronic infections, humans, rabbits, ribavirin, zoonoses, France

## Abstract

Hepatitis E virus (HEV) has been detected in rabbits, but whether rabbit HEV strains can be transmitted to humans is not known. Of 919 HEV-infected patients in France during 2015–2016, five were infected with a rabbit HEV strain. None of the patients had direct contact with rabbits, suggesting foodborne or waterborne infections.

Reports of hepatitis E virus (HEV) infections in humans and animals are becoming more frequent. HEV is a member of the family *Hepeviridae.* HEV strains that infect humans (HEV1, HEV2, HEV3, HEV4, and HEV7) belong to the genus *Orthohepevirus* (*1*). In industrialized countries, HEV transmission is mainly zoonotic, and the most prevalent genotype is HEV3. This genotype is transmitted mainly by direct contact with infected pigs, eating contaminated food products, or the environment (*2*). Genotype 3 includes 3 clades, 2 (3-efg and 3-abchij) of which are found in humans and pigs and 1 (3-ra) of which is found in rabbits (*3*). HEV3-ra has a 93-nt insertion in the X domain of the genome (*4*). This virus has been identified in both farmed and wild rabbits worldwide (*4*) and a pet rabbit (*5*).

HEV3 infections are generally asymptomatic and self-limiting, but symptomatic acute hepatitis develops in some patients, mostly older men. Fulminant hepatitis can occur in patients with underlying liver disease, and HEV3 infections can become chronic in immunocompromised patients, such as recipients of solid-organ transplants, persons with hematologic diseases, and patients infected with HIV (*2*). Although only 1 case of infection with human HEV3-ra has been identified (*6*), the contribution of HEV3-ra to human infection remains uncertain.

## The Study

The French National Reference Center for HEV (Paris, France) analyzed 919 HEV strains obtained from patients in France infected during 2015–2016. Strains were obtained in hospitals (90%) or private medical laboratories (10%). A total of 20% of the strains were obtained from immunocompromised patients.

We detected HEV RNA by using a reverse transcription PCR (RT-PCR) 15189 accredited by the International Organization of Standardization (Geneva, Switzerland) (*7*). We used a nested RT-PCR to amplify a 345-nt sequence within HEV open reading frame 2 as described (*8*). To amplify a 365-nt fragment within the X domain, we performed a nested RT-PCR with outer primers 2600-DOM-X-S (5′-TAYCGRGARACYTGYTCCCG-3′) and 3050-DOM-X-AS (5′-ACATCRACATCCCCCTGYTGTATRGA-3′) and inner primers 2685-DOM-X-S (5′-AGYTTTGAYGCCTGGG-3′) and 3050-DOM-X-AS. Phylogenetic analyses were performed by using neighbor-joining methods, a bootstrap of 1,000 replicates, and MEGA version 7.0 software (http://www.megasoftware.net/).

Among the 919 patients, 904 were infected with a genotype 3 strain. The subtype infecting 75 (8.1%) of these HEV3-infected patients could not be determined; 302 (32.9%) patients were infected with clade HEV3-abchij, 522 (56.8%) were infected with clade HEV3-efg, and 5 (0.5%) were infected with clade HEV3-ra (GenBank accession nos. KY611812–KY611816) (Figure). All 5 HEV3-ra strains had an insertion in the X domain of the genome (GenBank accession nos. KY825957–KY825961).

**Figure:**
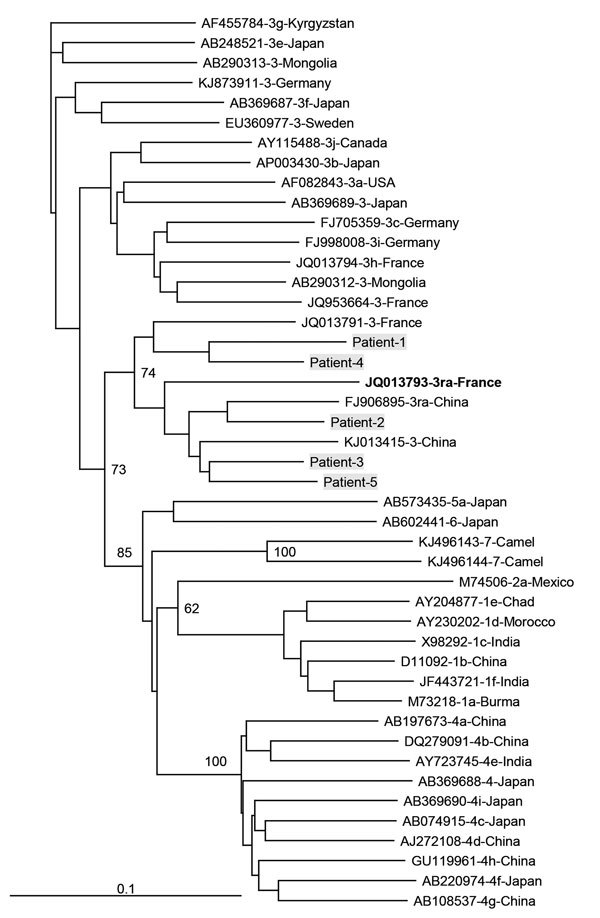
Phylogenetic tree for a 347-nt sequence within open reading frame 2 of hepatitis E viruses, France. Genetic distances were calculated by using the Kimura 2-parameter method, and the tree was plotted using the by the neighbor-joining method. Values along branches are bootstrap values acquired after 100 replications. Virus sequences obtained from patients in this study (gray shading) were compared with reference sequences of subtypes 3 viruses according to the proposal of Smith et al. (*3*). GenBank accession numbers, genotypes, and countries of origin are listed. Reference sequence in bold is from a hepatitis E virus genotype 3-ra isolated from a human. Scale bar indicates nucleotide substitutions per site.

All 5 infected patients were men (median age 52 years, range 38–64 years). None of these men had epidemiologic links to HEV or had traveled abroad; 2 lived in northern France, and 3 lived in southern France. One infected patient was immunocompetent and 4 were immunocompromised. The immunocompetent patient had alcoholic cirrhosis and decompensation of his cirrhosis because of the HEV infection. He cleared the HEV infection spontaneously.

All 4 immunocompromised patients were asymptomatic: 2 were solid-organ transplant recipients, and 2 had hematologic malignancies that were being treated with chemotherapy. Levels of alanine aminotransferase for these 4 patients were persistently high, and plasma HEV RNA was still detected 3 months after the initial evaluation, which indicated a chronic infection (*9*). Three patients were given ribavirin therapy for 3 months. The patients with hematologic malignancies eliminated the virus, but the kidney transplant recipient had a relapse when treatment was stopped.

The source of infection was unclear because all 5 patients reported they had no direct contact with rabbits. Three patients lived in rural areas, but none recalled any contact with wild or farmed animals. None of them was a hunter. Two patients reported they had eaten rabbit products, but the products were always well-cooked. All patients regularly ate various pork products, and 3 frequently ate raw shellfish (oysters, mussels, or scallops). Two drank tap water, and 3 drank only bottled water. One patient had a vegetable garden (Table).

**Table:** Characteristics of 5 men infected with rabbit hepatitis E virus, France

Characteristic	Patient				
	1	2	3	4	5
Age, y	52	38	64	50	62
Location	Southern (urban)	Northern (urban)	Northern (rural)	Southern (rural)	Southern (rural)
Underlying disease or condition	Alcoholic cirrhosis	Kidney transplant	Myeloma	Lymphoma	Heart transplant
Symptomatic acute hepatitis E	Yes	No	No	No	No
Contact with wild animals	No	No	No	No	No
Consumed rabbit	No	Yes	Yes	No	No
Consumed pork	Yes	Yes	Yes	Yes	Yes
Consumed shellfish	No	No	Yes	Yes	Yes
Type of drinking water	Tap	Bottled	Bottled	Bottled	Tap
Vegetable garden	No	No	No	Yes	No

## Conclusions

HEV3-ra can infect humans and its pathogenesis is similar to that of other HEV3 subtypes. There have been frequent reports of autochthonous HEV3 infections in several industrialized countries, particularly France (*10*). The HEV3 subtypes responsible are usually similar to those found in swine. These subtypes belong to clades 3-efg and 3-abchij (*11*,*12*). Their distribution among 919 symptomatic cases (3c, 26.7%; 3e, 2%; and 3f, 47%) in our study was similar to that reported for asymptomatic blood donors in France (*13*). However, HEV3-ra strains were not detected in blood donors, probably because of a small sample size.

The rarity of HEV3-ra infections in humans could be the result of fewer persons eating rabbit than pork products (*14*). In addition, we found that 80% of the HEV3-ra strains were obtained from immunocompromised patients. However, this population represents only 20% of the HEV-infected patients characterized by our laboratory. This HEV3 subtype could be less infectious than other subtypes for humans, but additional studies are needed to verify this hypothesis.

We could not identify the route by which our patients became infected with HEV3-ra. None had any direct contact with rabbits; 2 had eaten well-cooked rabbit. Data from a recent nationwide study in France suggested that waterborne transmission might play a role in HEV epidemiology (*14*). This type of transmission might be the way the patients in our study became infected because we have detected HEV3-ra in environmental samples (F. Abravanel, unpub. data).

To our knowledge, clinical manifestations associated with HEV3-ra have not been reported. We found that HEV3-ra infections caused severe decompensation in a patient with alcoholic cirrhosis. Chronic infections developed in the 4 immunocompromised patients. Patients in industrialized countries infected with other HEV3 subtypes also showed similar clinical findings (*1*). We also found that ribavirin had an antiviral effect on HEV3-ra. Ribavirin eliminated the virus in 2 of the 3 patients given this drug. This finding is consistent with that of a multicenter study, which reported that 78% of persons with chronic HEV3 infections were successfully treated with this drug (*15*).

Our findings emphasize the zoonotic risk for HEV3-ra and expand the spectrum of potential sources of human infection. The route by which HEV3-ra is transmitted to humans needs to be investigated. Longitudinal study of HEV diversity is also needed to assess trends over time.
